# POEMS Syndrome Without a Detectable Monoclonal Peak: The Critical Role of VEGF and Bone Marrow Biopsy in Diagnosis

**DOI:** 10.1155/crh/5530850

**Published:** 2025-09-18

**Authors:** Paul J. Pecorin, Max Melchioris A., Guy Olson, Emily Flammersfeld, Marwah Al Tekreeti, Patrick Atisha

**Affiliations:** ^1^Department of Internal Medicine, Rush University Medical Center, Chicago, Illinois, USA; ^2^Department of Neurological Sciences, Rush University Medical Center, Chicago, Illinois, USA; ^3^Department of Pathology, Rush University Medical Center, Chicago, Illinois, USA

**Keywords:** monoclonal plasma cell disorder, POEMS syndrome, VEGF

## Abstract

Polyneuropathy, organomegaly, endocrinopathy, monoclonal plasma cell disorder, skin changes (POEMS) syndrome is a rare disorder that is frequently misdiagnosed due to its heterogeneous presentation and overlap with chronic inflammatory demyelinating polyneuropathy (CIDP). Diagnosis requires the presence of polyneuropathy and a monoclonal plasma cell disorder, along with additional major and minor criteria. We present a 73-year-old woman with progressive weakness, volume overload, and weight loss, initially diagnosed with CIDP. Despite IVIG therapy, her symptoms worsened. Notably, no monoclonal peak was detected on serum protein electrophoresis (SPEP) or immunofixation, complicating the diagnosis. However, markedly elevated vascular endothelial growth factor (VEGF) levels (11.245 pg/mL) and bone marrow biopsy findings of a monoclonal plasma cell disorder confirmed POEMS syndrome. She also developed multiple thromboembolic events, highlighting the syndrome's prothrombotic nature. This case underscores the importance of maintaining high suspicion for POEMS syndrome in the setting of undifferentiated polyneuropathy, even in the absence of a monoclonal peak on SPEP. VEGF measurement and bone marrow biopsy are crucial for diagnosis in such cases. Early recognition and treatment, including plasma cell-directed therapy and anticoagulation, are essential to improving patient outcomes and preventing irreversible complications.

## 1. Introduction

Polyneuropathy, organomegaly, endocrinopathy, monoclonal plasma cell disorder, skin changes (POEMS) syndrome is a rare paraneoplastic syndrome linked to an underlying plasma cell dyscrasia. Its diagnosis requires both peripheral polyneuropathy as well as a monoclonal plasma cell disorder, along with a minimum of one additional major and one minor criterion. Major criteria include sclerotic bone lesions, Castleman disease, and elevated VEGF levels. Minor criteria consist of organomegaly, endocrinopathy, skin changes, papilledema, extravascular volume overload, and thrombocytosis or polycythemia [1].

Given its wide range of clinical presentations, POEMS syndrome is often difficult to diagnose and frequently misclassified as CIDP. However, hallmark features of thrombocytosis, elevated VEGF levels, and prominent axonal degeneration on EMG lend credence to a diagnosis of POEMS [2–4]. Although identification of a monoclonal protein, typically via SPEP or IFE, remains a critical diagnostic step, in one cohort, it was absent in 11% of patients [5].

Here, we share a case of an atypical presentation of POEMS syndrome with the absence of a monoclonal peak on serum and urine studies, which was initially diagnosed as CIDP. Diagnosis of POEMS syndrome was confirmed with VEGF and bone marrow biopsy, demonstrating the importance of these tests in appropriate diagnosis and treatment.

## 2. Case

A 73-year-old woman with no known family or genetic history and a personal history of coronary artery disease, stroke without residual deficits, and hypertension presented with progressive bilateral lower extremity weakness, lower extremity swelling, and subacute cognitive decline.

Her symptoms began 8 months prior to admission when she developed progressive lower extremity weakness. Several weeks later, she noted increasing lower extremity edema. Over the ensuing months, her mobility deteriorated—from walking with a cane to requiring a walker and eventually a wheelchair. She also experienced an unintentional 20-pound weight loss. One month before admission, she was evaluated at an outside emergency department, where magnetic resonance imaging (MRI) of her spine revealed moderate canal narrowing without compression.

On admission, physical examination demonstrated 3/5 right grip strength and finger abduction, 4/5 left grip strength and finger abduction, 2/5 bilateral hip flexion, 1/5 bilateral knee extension and flexion, 0/5 bilateral foot dorsiflexion and plantar flexion, and 2+ pitting edema to the knees. Initial laboratory evaluation was notable for hemoglobin 15.2 g/dL, leukocyte count 5.46 × 10^9^/L, platelet count 708 × 10^9^/L, vitamin B12 214 pg/mL, TSH 1.6 μIU/mL, HbA1c 6.5%, negative HIV, copper 73 μg/dL, zinc 59 μg/dL, iron 45 μg/dL, TIBC 231 μg/dL, and iron saturation 19%. SPEP showed elevated lambda light chains (17.05 mg/dL) with a suppressed kappa/lambda ratio (0.18), but no paraproteins or monoclonal peaks on immunofixation. Given the progressive, symmetric, predominantly motor neuropathy with associated edema and a monoclonal gammopathy, the differential diagnosis included POEMS syndrome, chronic inflammatory demyelinating polyneuropathy (CIDP), amyloidosis, and nutritional deficiencies. VEGF was sent, and the patient was admitted for further evaluation.

Imaging revealed mild degenerative changes without significant spinal canal stenosis on MRI of the spine. MRI of the brain showed no acute pathology. Electromyography (EMG) and nerve conduction study (NCS) showed severe demyelinating polyneuropathy with prominent axon loss, consistent with a primary demyelinating pathology (Table 1, Supporting Figure 1).

Despite venous thromboembolism (VTE) prophylaxis with subcutaneous heparin, on hospital Day 3, she was found to have multiple thromboembolic events. These included a deep vein thrombosis (DVT) in the left proximal mid-posterior tibial vein, bilateral proximal femoral vein DVTs, and subsegmental pulmonary embolism (PE) in the right middle and lower lobes, prompting initiation of therapeutic anticoagulation. PEs were incidentally identified on CT abdomen and pelvis which did not show organomegaly.

Cerebrospinal fluid (CSF) analysis revealed an elevated protein level of 220 mg/dL, glucose of 60 mg/dL, and white blood cell count of 2 cells/μL. Cytology was negative. She was started on intravenous immunoglobulin (IVIG) at 0.4 g/kg for 5 days for presumed CIDP, but corticosteroids were deferred due to concurrent influenza infection.

**Figure 1:**
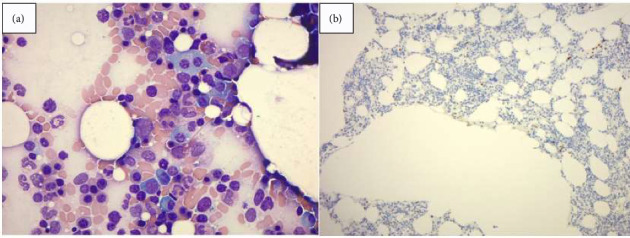
(a) Bone marrow aspirate demonstrating a plasma cell neoplasm with mild atypia with 6% involvement. (b) Cyclin D1 immunohistochemical staining demonstrating 3% involvement of plasma cell neoplasm.

Despite IVIG, her symptoms did not significantly improve; in fact, she reported subjective worsening of upper extremity weakness. On hospital Day 10, the VEGF study resulted and was found markedly elevated at 11.245 pg/mL. Given the elevation of VEGF and lack of clinical response to IVIG, bone marrow biopsy was completed on hospital Day 12 which revealed a plasma cell neoplasm with mild atypia involving 6% of the sample in aspirate smear (Figure 1(a)) and 3% on cyclin D1 immunostaining (Figure 1(b)). Flow cytometry demonstrated 1% plasma cells with lambda light chain predominance, confirming the presence of a monoclonal plasma cell disorder.

Based on her clinical presentation—including severe demyelinating polyneuropathy, monoclonal plasma cell disorder (mandatory major criteria), elevated VEGF (additional major criteria), extravascular volume overload, and thrombocytosis (minor criteria)—she was diagnosed with POEMS syndrome. She was transferred to the hematology service and initiated on weekly bortezomib (1.3 mg/m^2^) and dexamethasone (40 mg for four days per cycle), with plans to add daratumumab and lenalidomide in the outpatient setting.

After completing her first month-long cycle of treatment, she reported some subjective improvement in upper extremity strength, although her neurological examination remained largely unchanged.

This case report was prepared in accordance with the CAse REport (CARE) guidelines to ensure comprehensive and transparent reporting (https://www.care-statement.org).

## 3. Discussion

POEMS syndrome remains a diagnostic challenge due to its variable presentation and overlap with other demyelinating neuropathies, such as CIDP. This case highlights the importance of a high index of suspicion for POEMS syndrome in patients presenting with progressive polyneuropathy, particularly when accompanied by systemic features such as thrombocytosis, volume overload, and weight loss.

A critical point of separation between POEMS syndrome and CIDP is the presence of multisystem manifestations beyond neuropathy. While CIDP typically presents as an isolated neuropathy, POEMS is accompanied by multisystem involvement including endocrinopathies, organomegaly, sclerotic bone lesions, and hematologic derangements such as thrombocytosis and polycythemia [1]. The presence of thrombocytosis and extravascular volume overload in our patient raised suspicion for POEMS syndrome early in her course, particularly given the lack of response to IVIG therapy. Additionally, VEGF elevation has emerged as a critical biomarker for POEMS syndrome, aiding in the differentiation from CIDP [4]. In this case, the markedly elevated VEGF level (11.245 pg/mL) was instrumental in confirming the diagnosis.

The absence of a monoclonal protein on serum immunofixation posed a diagnostic challenge. Although monoclonal proteins are detected in most cases of POEMS syndrome, their absence does not exclude the diagnosis. The 2023 American Journal of Hematology update emphasizes the low clonal burden often seen in POEMS, which may account for this finding [1]. In a cohort of 392 patients, approximately 11% lacked detectable monoclonal protein [5]. In clinical practice, this can lead to premature dismissal of the diagnosis, delaying essential treatment. Our case illustrates the importance of pursuing additional diagnostic modalities, including bone marrow biopsy and flow cytometry, which ultimately revealed a monoclonal plasma cell disorder. VEGF measurement and marrow evaluation should therefore be strongly considered in patients with high clinical suspicion, even in the absence of a monoclonal peak on serum studies.

Thrombotic complications are commonly recognized in POEMS, with recent literature citing a 30% incidence of events such as DVT and PE [6]. Risk factors include thrombocytosis, volume overload, splenomegaly, and erythrocytosis, several of which were seen in our patient. This case supports the recommendation for early thromboprophylaxis via aspirin or anticoagulation in POEMS syndrome, even before plasma cell-directed therapy is initiated [1].

Early diagnosis and appropriate therapy are crucial in POEMS syndrome, as delayed treatment is associated with increased morbidity and functional decline. The mainstay of therapy includes radiotherapy for localized disease and autologous stem cell transplantation (ASCT) for appropriate candidates. Additional therapeutic options include corticosteroids, alkylating agents, and lenalidomide. Bortezomib and thalidomide can also be considered, but cautiously as these have potential to worsen neuropathy [1, 7]. In this case, the patient was initiated on a combination of bortezomib and dexamethasone, with plans to consider daratumumab and lenalidomide as an outpatient due to poor ASCT candidacy. Although her neurological deficits persisted at 1-month follow-up, subjective improvement in upper extremity strength suggests potential for ongoing recovery with continued therapy.

This case has several strengths, including early recognition of systemic features and prompt evaluation for POEMS syndrome, even in the absence of a monoclonal protein on initial serum studies. Comprehensive diagnostic workup, including VEGF testing and bone marrow biopsy, enabled timely diagnosis and initiation of appropriate therapy.

However, there are notable limitations. Limited longitudinal follow-up data beyond the initial hospitalization constrain assessment of the patient's long-term neurological recovery and response to therapy. Additionally, a lymph node biopsy was not performed, which may have further supported the diagnosis by identifying Castleman disease, a known associated feature of POEMS syndrome. These gaps highlight the challenges in fully characterizing rare multisystem syndromes within a single hospitalization and underscore the importance of ongoing outpatient follow-up and multidisciplinary care.

## 4. Conclusion

This case highlights the diagnostic complexity of POEMS syndrome, particularly when monoclonal protein is absent on serum studies. The markedly elevated VEGF level and bone marrow biopsy were crucial in confirming the diagnosis, underscoring the importance of comprehensive evaluation in patients with progressive polyneuropathy and systemic features. Clinicians should be aware that a negative SPEP does not exclude POEMS syndrome, and additional diagnostic tools—including VEGF measurement and bone marrow biopsy—can be pivotal in securing the diagnosis. Additionally, this case reinforces the significant thrombotic risk associated with POEMS syndrome and the potential role of early anticoagulation to prevent complications. Early recognition and treatment are essential to prevent irreversible complications, highlighting the importance of maintaining a high index of suspicion and prompt diagnostic evaluation.

## Figures and Tables

**Table 1:** Nerve conduction study (NCS) and electromyography (EMG) findings.

Test	Nerve/muscle	Site	Latency (ms)	Amplitude (mV)	Velocity (m/s)	Findings
Sensory NCS	Median (R)	Digit II—wrist	No response (NR)	NR	NR	NR
	Ulnar (R)	Digit V—wrist	NR	NR	NR	NR
	Sural (R)	Ankle—calf	NR	NR	NR	NR

Motor NCS	Median (R)	Wrist—abductor pollicis brevis (APB)	6.46	2.6	—	Prolonged latency, reduced amplitude
	Median (R)	Elbow—APB	17.34	1.8	17	Prolonged latency, reduced amplitude, slowed velocity
	Median (L)	Wrist—APB	7.03	2.3	—	Prolonged latency, reduced amplitude
	Median (L)	Elbow—APB	17.08	0.6	23	Prolonged latency, reduced amplitude, slowed velocity
	Ulnar (R)	Wrist—abductor digiti minimi (ADM)	8.91	0.3	—	Prolonged latency, low amplitude
	Ulnar (R)	B. elbow—ADM	NR	NR	NR	NR
	Peroneal (R)	Ankle—extensor digitorum brevis	NR	NR	NR	NR
	Tibial (R)	Ankle—abductor hallucis	NR	NR	NR	NR

F-wave	Median (R)	APB	NR	—	—	NR

EMG	Tibialis anterior (R)	L4-L5	3+ Fib/PSW	No activity	—	Severe denervation

*Note:* EMG and NCS demonstrating severe demyelinating polyneuropathy. Significant slowing in the upper extremity is present, most consistent with a primary demyelinating neuropathy. See supporting Figure 1 for additional interpretation.

## Supporting Information

Supporting Figure 1: Detailed interpretation of EMG and NCS data is provided in Table 1 demonstrating severe demyelinating polyneuropathy with prominent axon loss, consistent with a primary demyelinating pathology.

## References

[1] Dispenzieri A. (2023). POEMS Syndrome: Update on Diagnosis, risk-stratification, and Management. *American Journal of Hematology*.

[2] Naddaf E., Dispenzieri A., Mandrekar J., Mauermann M. L. (2015). Thrombocytosis Distinguishes POEMS Syndrome from Chronic Inflammatory Demyelinating Polyneuropathy. *Muscle & Nerve*.

[3] Pihan M., Keddie S., D’Sa S. (2018). Raised VEGF: High Sensitivity and Specificity in the Diagnosis of POEMS Syndrome. *Neurology Neuroimmunology & Neuroinflammation*.

[4] Brown R., Ginsberg L. (2019). POEMS Syndrome: Clinical Update. *Journal of Neurology*.

[5] Suichi T., Misawa S., Beppu M. (2019). Prevalence, Clinical Profiles, and Prognosis of POEMS Syndrome in Japanese Nationwide Survey. *Neurology*.

[6] Sayar Z., Weatherill A., Keddie S. (2020). High Rates of Venous and Arterial Thrombotic Events in Patients with POEMS Syndrome: Results From the UCLH (UK) POEMS Registry. *Blood Advances*.

[7] Nozza A., Terenghi F., Gallia F. (2017). Lenalidomide and Dexamethasone in Patients with POEMS Syndrome: Results of a Prospective, Open-Label Trial. *British Journal of Haematology*.

