# Supplementary material for: POEMS Syndrome Without a Detectable Monoclonal Peak: The Critical Role of VEGF and Bone Marrow Biopsy in Diagnosis
Source: Case Rep Hematol. 2025 Sep 18;2025:5530850. doi: 10.1155/crh/5530850 (PMC12463516; doi:10.1155/crh/5530850)
Supplement: Supporting Information — Additional supporting information can be found online in the Supporting Information section. [file 5530850.f1.docx]

**Supplemental Figure 1: EMG/NCS Interpretation**

**Sensory NCS:** The patient had no response in the right median (wrist), right ulnar (wrist), and right sural (calf) nerves for latency (milliseconds, ms), amplitude (microvolts, µV), and velocity (meters per second, m/s). The absence of response in latency and velocity is consistent with demyelination, while the lack of amplitude response is indicative of axonal injury.

**Motor NCS**

- **Right Median Nerve (APB):**
  - Latency at the wrist is elevated at **6.46 ms**, compared to the reference value of **≤ 4.4 ms**, consistent with demyelination.
  - Conduction velocity is **40 m/s**, below the reference value of **≥ 49 m/s**, also consistent with demyelination.
  - Amplitude at the wrist is low at **2.6 mV**, compared to the reference value of **≥ 4.0 mV**, consistent with axonal injury.
- **Left Median Nerve (APB):**
  - Latency at the wrist is elevated at **7.03 ms**, compared to the reference value of **≤ 4.4 ms**, consistent with demyelination.
  - Conduction velocity is **39 m/s**, below the reference value of **≥ 49 m/s**, also consistent with demyelination.
  - Amplitude at the wrist is low at **2.3 mV**, compared to the reference value of **≥ 4.0 mV**, consistent with axonal injury.
- **Right Ulnar Nerve (ADM):**
  - Latency at the wrist is elevated at **8.91 ms**, compared to the reference value of **≤ 3.5 ms**, consistent with demyelination.
  - Amplitude at the wrist is reduced to **2.1 mV**, compared to the reference value of **≥ 5.0 mV**, consistent with axonal injury.
  - No response at the elbow for latency and amplitude, consistent with severe demyelination and axonal injury.
- **Right Peroneal (ankle) and Right Tibial (ankle) Nerves:**
  - No response for amplitude (mV) and latency (ms), consistent with severe demyelination and axonal injury.

**F-wave Response:**

- **Right Median (APB):** No response, consistent with proximal nerve injury.

**EMG Findings:**

- **Right Tibialis Anterior:**
  - Increased **insertional activity (+3)**.
  - Presence of **fibrillation potentials** and **positive sharp waves**, consistent with active denervation, as evidenced by spontaneous depolarization due to lack of neural input.

**Conclusion:** Findings are consistent with a severe, generalized peripheral neuropathy demonstrating both axonal and demyelinating features, involving sensory and motor fibers. This interpretation is based on established electrodiagnostic criteria for peripheral nerve disorders and aligns with previously described patterns in POEMS syndrome and other acquired neuropathies. Differential considerations include chronic inflammatory demyelinating polyneuropathy (CIDP), Guillain-Barré syndrome (GBS), diabetic polyneuropathy, and toxic/metabolic etiologies [1,2,3].

1. **Preston DC, Shapiro BE.** Electromyography and Neuromuscular Disorders: Clinical-Electrophysiologic Correlations. 4th ed. Elsevier; 2021.

2. **Dispenzieri A.** POEMS Syndrome: 2023 Update on Diagnosis, Risk-Stratification, and Management. *Am J Hematol*. 2023;98(6):847-860. doi:10.1002/ajh.26880

3. **American Association of Neuromuscular & Electrodiagnostic Medicine (AANEM).** Practice Topic: Criteria for the Diagnosis of Distal Symmetric Polyneuropathy. AANEM; 2022. Available at: <https://www.aanem.org> (Accessed May 18, 2025).
